# Trajectories of maternal sleep problems before and after childbirth: a longitudinal population-based study

**DOI:** 10.1186/s12884-015-0577-1

**Published:** 2015-06-02

**Authors:** Børge Sivertsen, Mari Hysing, Signe K. Dørheim, Malin Eberhard-Gran

**Affiliations:** Division of Mental Health, Norwegian Institute of Public Health, Kalfarveien 31, 5018 Bergen, Norway; The Regional Centre for Child and Youth Mental Health and Child Welfare, Uni Research Health, Bergen, P.O. Box 7810, N-5020 Bergen, Norway; Department of Psychiatry, Helse Fonna HF, P.O. Box 2170, N-5504 Haugesund, Norway; Division of Psychiatry, Stavanger University Hospital, PO Box 8100, Stavanger, NO-4068 Norway; Health Services Research Center, Akershus University Hospital, Lørenskog, Norway; Institute of Clinical Medicine, Campus Ahus, University of Oslo, Lørenskog, Norway

**Keywords:** Sleep problems, Insomnia, Pregnancy, Longitudinal, Epidemiology

## Abstract

**Background:**

Sleep problems are common during pregnancy and in the postnatal period, but there is still a lack of longitudinal population-based studies assessing the quantity and quality of sleep in these women. The aim of the current study was to examine the natural development and stability of insomnia and short sleep duration in women from pregnancy to two years postpartum.

**Methods:**

This was a longitudinal cohort study (the Akershus Birth Cohort Study) of 1480 healthy women, who completed three comprehensive health surveys, at week 32 of pregnancy, week 8 postpartum and year 2 postpartum. The survey was composed of the following validated questionnaires: the Bergen Insomnia Scale, the Pittsburgh Sleep Quality Index and the Edinburgh Postnatal Depression Scale. Differences in sleep characteristics between the three assessment points were compared using Analyses of Variance with repeated measures, and logistic regression analyses were used to examine the stability of sleep variables.

**Results:**

One thousand four hundred and eighty women completed all three surveys, and the mean age at birth was 30.7 (+/−4.9). The prevalence of insomnia remained stable at 60 % at the first two time periods, and remained high at 41 % at year 2 postpartum. The mean sleep duration at the three time periods was 7 h 16 min, 6 h 31 min, and 6 h 52 min, respectively. Concurrent maternal depression could not explain the stability of sleep problems from during and immediately after pregnancy, to sleep problems 2 years postpartum.

**Conclusion:**

Both insomnia and short sleep duration were found to be very common both before and after pregnancy.

## Background

Insomnia is one the most common health complaints in the general population, and is associated with substantial individual [1] and societal consequences [2]. With rising prevalence rates over the last decade [3], it is now estimated that approximately 10–15 % of the adult population fulfills the diagnostic criteria for insomnia disorder [3]. Women experience more insomnia than men [4], a gender pattern which emerges in late adolescence [5]. Pregnancy and the postnatal period may be an especially vulnerable period for the development of insomnia and other sleep disturbances in women. This is not surprising given the many physical and physiologic changes during pregnancy. Similarly, nighttime feeding and the frequent nocturnal awakenings among infants are important factors for understanding sleep deficits and reduced sleep quality among mothers in the postnatal period [6].

Short sleep duration and symptoms of insomnia may thus be regarded as common phenomena during pregnancy and the postnatal period. Previous findings show that the first trimester is typically characterized by an increase in total sleep time and daytime sleepiness [7], whereas the majority of pregnant women experience reduced sleep quality and more nocturnal awakenings later in pregnancy [8–13], and immediately after birth [14–16]. However, these findings are primarily based on small-scale studies, and there is still a lack of population-based studies assessing the quantity and quality of the sleep of women during pregnancy and postpartum. Furthermore, to the best of our knowledge, no studies have investigated whether such sleep problems continue beyond the first few months postpartum. A longitudinal population-based study from Norway recently showed that nocturnal awakenings become less frequent when toddlers reach 18 months of age [6], suggesting that also the mothers sleep may improve at this stage compared to the weeks and months immediately after the birth. On the other hand, insomnia often takes a chronic course [17], and it has yet to be investigated whether sleep problems emerging during pregnancy continue beyond the infant stage and into toddlerhood.

Moreover, previous findings from the same data that is used in the current study have shown that depressive symptoms and sleep problems are closely interrelated, both in late pregnancy (week 32) and in the postnatal period (8 weeks postpartum) [18–20]. Therefore, to better understand the extent to which sleep problems endure over time in this population, depressive symptoms need to be accounted for when examining the chronicity of sleep problems from the pre- to postnatal period.

Based on the above considerations, the aims of the current study were 1) to describe the natural development and stability of insomnia and short sleep duration from pregnancy to 8 weeks and 2 years postpartum, and 2) to examine to what extent the predictive effect of early sleep problems during pregnancy on maternal insomnia in toddlerhood may be explained by postnatal depressive symptoms.

## Methods

### Study population and design

**Fig. 1:**
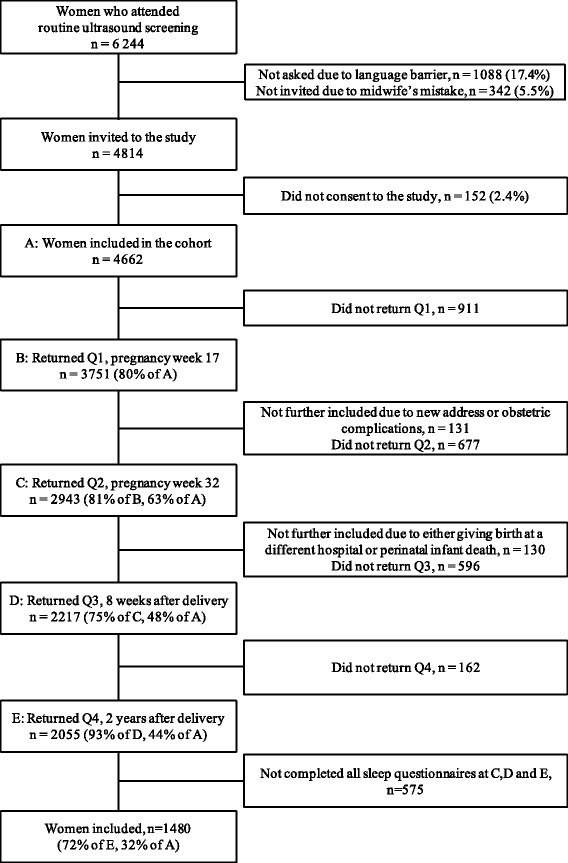
Flow chart of study

The Akershus Birth Cohort is a longitudinal questionnaire study targeted at all women giving birth at Akershus University Hospital in Norway. The hospital serves a population of 350,000 from urban and rural areas. All women scheduled to give birth at the hospital were approached in gestational week 17, when they underwent routine fetal ultrasound. Women were included if they gave consent to participate and were able to complete a questionnaire in Norwegian. The recruitment lasted from November 2008 until April 2010. Consenting women were handed a questionnaire at gestational week 17, and thereafter received a questionnaire by mail at week 32 of pregnancy, 8 weeks after delivery, and 2 years after delivery. In total, 2943 women returned second questionnaire, 2217 women returned the third questionnaire and 2055 women returned the fourth questionnaire. Due to 575 women not completing the sleep questionnaires at all three time points, the final study sample in the current study comprised 1480 women, yielding a participation rate of 32 % of the 4662 women who originally consented to participate, and 72 % of the women who returned the fourth questionnaire. See Fig. 1 for a flow chart of participants. Note that the sample sizes at the different time points may deviate somewhat from previous AHUS publications due to small changes in the latest quality-assured data file released for research.

### Instruments

#### Demographical and clinical information

Demographic information collected at week 17 included maternal age, marital status (married or cohabitating versus single/widowed/divorced), number of previous children (parity), and level of education (elementary school, completed high school, or higher education). Body-mass index was assessed both at week 17 and week 32 and was calculated from weight (kg) divided by squared height (m2). We also collected data on chronic hypertension and diabetes.

#### Sleep variables

The Bergen Insomnia Scale (BIS) [21] was used to assess insomnia at all 3 time periods. The BIS includes 6 items that correspond to the diagnostic criteria for insomnia in DSM-IV-TR. Each item is scored using a scale from 0 to 7, where the respondents specify the frequency of the various insomnia symptoms in terms of days per week. The first four items assess sleep impairment (DSM-IV-TR criterion A for insomnia), and the last two items measure daytime sleepiness or tiredness (affecting school/work or private life) and dissatisfaction with sleep (DSM-IV-TR criterion B for insomnia). The BIS provides both a total score on a continuous scale and a dichotomous score for the presence or absence of insomnia syndrome. The BIS has demonstrated good psychometric properties [21].

Three questions from the Pittsburgh Sleep Quality Index (PSQI) [22] were included at all 3 time periods. These assessed average time they went to bed, average wake up time in the morning and average total sleep duration per night during the previous month. From these data, habitual sleep efficiency (time asleep divided by total time spent in bed) was calculated, and a joint variable encompassing sleep onset latency (SOL) and wake after sleep onset (WASO) was also calculated subtracting reported sleep duration from time in bed. For purposes of the present study, a variable of short sleep duration was also calculated, defined as sleeping less than 7 h.

#### Depressive symptoms

The Edinburgh Postnatal Depression Scale (EPDS) [23, 24] was used to measure depressive symptoms at all three time periods. The EPDS is a 10-item self-rating questionnaire developed to screen for depression in the postpartum period; it addresses symptoms present in the last seven days. The scale also has good psychometric properties during pregnancy [25]. Each question has four alternative answers, scoring 0 to 3, for a maximum score of 30. A cut off of 10 or above was found to have good psychometric properties for a diagnosis of depression among Norwegian postpartum women, and this cut off has also been used in previous studies of pregnant women [26].

### Statistics

All analyses were performed using the SPSS statistical software package, version 22 (SPSS Inc., Chicago, IL, USA). Analyses of Variance (ANOVA) with repeated measures with Greenhouse-Geisser correction and Bonferroni post hoc test were used to test for time differences in sleep variables at the three time points. Within-group effect sizes (pooled SD) were calculated using Cohen’s *d* – formula. According to Cohens’ guidelines [27], these effect sizes should be interpreted with *d*s around .20 representing small effect sizes, *d*s of about .50 moderate effect sizes and *d*s above .80 large effect sizes. Negative binomial regression analyses were used to examine associations between sleep variables at T1 and T2, and subsequent maternal insomnia and short sleep duration at T3. Negative binomial regressions (producing relative risk (RR)) were used rather than more commonly used logistic regressions (producing odds-ratio (OR)), as ORs tend to overstate an effect size compared to RRs when the prevalence of the outcome of interest (in this case insomnia or short sleep duration) is high [28]. Both crude/unadjusted and adjusted analyses were conducted. Adjustment variables included maternal age, marital status, level of education, parity, BMI and depressive symptoms at all three time points.

### Ethics

All women asked to participate were given written information explaining the purpose of the study and informed that participation was voluntary. Informed consent was obtained from all participants. The study was approved by the Regional Committee for Ethics in Medical Research in Norway, approval number S-08013a.

## Results

### Sample characteristics

The longitudinal sample included 1480 women, with a mean age of 30.7 (SD = 4.9). The vast majority of women (97.9 %) were married or living with a partner, and a majority of the sample (72.6 %) also had an educational level beyond high school. The mean BMI was 24.5 (SD = 4.5), and 79.6 % reported that this was their first pregnancy (see Table 1 for details). As also detailed in Table 1, women who completed all assessments (responders) were older, more likely to be married/cohabitating, as well as have higher education, compared to women who did not complete all assessments (non-responders).

**Table 1:** Demographical and clinical characteristics of responders (all time points; n = 1480) and non-responders (only first time point/week 17)

	Responders		Non-responders		*P*-level
	%		%		
Parity					.02
Primipara	79.6 %		76.4 %		
Multipara	20.4 %		23.6 %		
Marital status					<.001
Married/cohabitating	97.9 %		95.3 %		
Single	2.1 %		4.7 %		
Maternal educational					<.001
Elementary	1.8 %		7.3 %		
High school	25.7 %		39.2 %		
Higher degree	72.6 %		52.5 %		
	Mean	S.D.	Mean	S.D.	
Age	31.6	4.5	30.3	5.1	<.001
Body mass index (BMI) (week 32)	24.5	4.5	24.7	5.0	.246

There were no significant differences on any of the sleep measures at T1 between women who had dropped out at either T2 or T3, and women who completed all waves. However, women dropping out after T1 had significantly more symptoms of depression than those who took part at all three time points (mean = 5.9 [SD 4.7] versus 4.9 [SD 3.9], *P* < .001. respectively.

### Sleep characteristics

The repeated measures ANOVA showed that the mean sleep duration differed significantly between the three time points. As detailed in Table 2, the mean sleep duration was reduced from 7 h 16 min at week 32 of pregnancy to 6 h 31 min at week 8 postpartum, but increased to 6 h 52 min at year 2 postpartum (all *P*s < .001). Similar patterns were observed for both SOL/WASO and sleep efficiency; whereas these sleep problems increased substantially in magnitude from T1 to T2, they decreased again at year 2 postpartum (see Table 2 for details).

**Table 2:** Sleep characteristics of included participants at the three time points, week 32 of pregnancy, and week 8 and year 2 postpartum

	Week 32 of pregnancy (Time 1)		Week 8 postpartum (Time 2)		Year 2 postpartum (Time 3)		Time 1 vs Time 2		Time 2 vs Time 3		Time 1 vs Time 3	
	Mean/%	(SD/n)	Mean/%	(SD/n)	Mean/%	(SD/n)	p-level*	Cohen’s d	*p-level*	Cohen’s *d*	*p-level*	Cohen’s *d*
Sleep duration variables												
Time in bed, hh:mm	8:42	(1:16)	8:51	(1:22)	7:44	(0:51)	.010	0.11	<.001	0.98	<.001	0.90
Sleep duration, hh:mm	7:16	(1:23)	6:31	(1:16)	6:52	(0:58)	<.001	0.57	<.001	0.31	<.001	0.34
<7 h, % (n)	27.4 %	(742)	49.8 %	(958)	35.2 %	(680)		-		-		-
7-8 h, % (n)	31.4 %	(852)	29.8 %	(573)	45.1 %	(871)		-		-		-
≥8 h, % (n)	41.2 %	(1117)	20.4 %	(393)	19.7 %	(381)		-		-		-
SOL/WASO, hh:mm	1:28	(1:18)	2:21	(1:21)	0:52	(0:53)	<.001	0.67	<.001	1.30	<.001	0.54
Sleep efficiency, %	83.6 %	(13.3)	74.2 %	(13.2)	89.0 %	(10.5)	<.001	0.71	<.001	1.24	<.001	0.45
Insomnia variables												
DSM-IV Insomnia, % (n)	60.8 %	(1778)	59.6 %	(1305)	40.8 %	(835)	1.00	-	<.001	-	<.001	-
Difficulties initiating sleep, days/week	2.69	(2.38)	1.45	(2.00)	1.61	(1.97)	<.001	0.56	0.42	0.08	<.001	0.49
Difficulties maintaining sleep, days/week	2.93	(2.36)	4.24	(2.76)	1.66	(2.12)	<.001	0.51	<.001	1.05	<.001	0.57
Early morning awakenings, days/week	2.27	(2.27)	1.25	(2.06)	1.16	(1.81)	<.001	0.47	0.62	0.05	<.001	0.54
Non-restorative sleep, days/week	3.56	(2.16)	3.70	(2.31)	3.55	(2.30)	0.17	0.06	0.16	0.07	1.00	0.00
Daytime impairment, days/week	2.28	(2.20)	1.76	(2.00)	1.83	(2.00)	<.001	0.25	0.66	0.03	<.001	0.21
Sleep dissatisfaction, days/week	3.50	(2.30)	2.98	(2.35)	2.91	(2.32)	<.001	0.22	1.00	0.03	<.001	0.26

*P-value (< .05) based on Bonferroni Post hoc tests from Repeated-Measures ANOVA

The prevalence of insomnia at week 32 of pregnancy and 8 week postpartum remained stable at around 60 %, while it decreased to 41 % at year 2 postpartum. As also detailed in Table 2, which of the specific insomnia symptoms were most common differed across the three assessment points. The DSM-IV-TR criterion B for insomnia (Sleep dissatisfaction and daytime impairment) was significantly more commonly reported during pregnancy (T1) compared to T2 and T3, whereas difficulties maintaining sleep was significantly more common at T2, compared to both T1 and T3. Reports of non-restorative sleep were similar across all assessment points, despite the large differences in sleep durations, wake times and sleep efficiencies between T1, T2 and T3. All other sleep insomnia parameters were improved at T3, compared both to T1 and T2.

### Trajectories of insomnia

**Fig. 2:**
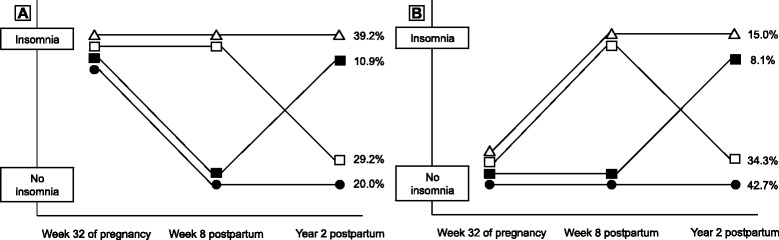
Trajectories of insomnia in mothers *with* (**a**) and *without* (**b**) insomnia at 32 weeks of pregnancy

Insomnia remained stable across the three assessment points. As detailed in Table 3, 68 % and 50 % of the pregnant women with insomnia at T1 still had insomnia at T2 and T3, respectively. Figure 2a depicts the trajectories of insomnia from week 32 of pregnancy through year 2 postpartum; 39 % of the women fulfilled the criteria for insomnia at all three assessment points, while 29 % reported insomnia at T1 and T2 only (not T3). Similarly, 43 % had had no insomnia at any of the three assessment points, while 34 % had insomnia only at week 8 postpartum (Fig. 2b).

**Table 3:** Prevalence of DSM-IV insomnia at week 8 and year 2 postpartum according to insomnia status at week 32 of pregnancy^a^

	Week 8 postpartum n^b^ (%)		Year 2 postpartum n^b^ (%)	
Week 32 of pregnancy, n (%)	No insomnia	Insomnia	No insomnia	Insomnia
No insomnia, 1145 (39.2 %)	407/787 (51.7 %)	380/787 (48.3 %)	518/690 (75.1 %)	172/690 (24.9 %)
Insomnia, 1778 (60.8 %)	372/1168 (31.8 %)	796/1168 (68.2 %)	495/990 (50.0 %)	495/990 (50.0 %)

^a^Data are based on all available cases at each time point

^b^Denominators represent available cases at the given time point

### Predictors of insomnia and short sleep duration at year 2 postpartum (T3)

A series of negative binomial regression analyses were conducted to examine to what extent different patterns of insomnia and short sleep duration at T1 and T2 predicted subsequent insomnia at T3 (year 2 postpartum). As detailed in Table 4, compared to not reporting insomnia at T1 and T2, having insomnia at both these points was associated with a relative risk of 3.58 for insomnia at T3. Adjusting for maternal age, education, marital status, parity, BMI and depressive symptoms (at all three time periods) did not reduce the association considerably (see Table 4 for details). Reporting insomnia at T1 only or T2 only was associated with RR of 2.21 and 1.92, respectively, and these associations also remained significant in the fully adjusted model. Similar patterns were observed for short sleep duration at T1 and T2 predicting insomnia at T3, although the magnitude of associations was smaller. Chronic short sleep (<7 h at T1 *and* T2) was associated with a relative risk of 1.56 for subsequent insomnia at T3, and also this association was only marginally reduced when adjusting for maternal age and depressive symptoms (RR = 1.40).

**Table 4:** Pattern of insomnia and short sleep duration (at T1 and T2) as risk factors for insomnia and short sleep duration at year 2 postpartum (T3)

		Insomnia at year 2 postpartum (T3).				Short sleep duration (<7 h) at year 2 postpartum (T3)			
		Unadjusted model		Adjusted model^#^		Unadjusted model		Adjusted model^#^	
	% (N)	RR	95 % CI	RR	95 % CI	RR	95 % CI	RR	95 % CI
Insomnia pattern									
No insomnia	20.8 % (407)	1.00	-	1.00	-	1.00	-	1.00	-
Insomnia T1 only	19.0 % (372)	2.21	1.60–3.06	2.06	1.40–3.03	1.12	0.86–1.45	0.90	0.65–1.23
Insomnia T2 only	19.4 % (380)	1.92	1.38–2.67	2.00	1.36–2.95	1.12	0.87–1.45	1.01	0.75–1.35
Chronic insomnia	40.7 % (796)	3.58	2.70–4.76	3.31	2.34–4.68	1.45	1.17–1.79	1.20	0.92–1.55
Pattern of short sleep duration*									
Normal/long sleep	43.3 % (700)	1.00	-	1.00	-	1.00	-	1.00	-
Short sleep T1 only	31.3 % (506)	1.39	1.15–1.68	1.37	1.10–1.71	1.35	1.08–1.70	1.29	1.00–1.68
Short sleep T2 only	8.4 % (136)	1.42	1.10–1.83	1.48	1.10–1.99	1.66	1.24–2.20	1.76	1.30–2.40
Chronic short sleep	16.9 % (273)	1.56	1.26–1.92	1.40	1.08–1.82	2.21	1.78–2.74	2.05	1.58–2.67

*Short sleep duration defined as < 7 h

^#^Adjusted for maternal age, education, marital status, parity, BMI (week 17 and 32), chronic hypertension, diabetes, and depressive symptoms (all three time periods)

Chronic insomnia (insomnia at both T1 *and* T2) was also a significant risk factor for short sleep duration at T3 in the crude analyses (RR = 1.45), but not after adjusting for confounders. Reporting insomnia at *only* T1 or T2 was not significantly associated with subsequent short sleep duration. As detailed in Table 4, previous short sleep duration significantly increased at the risk for reporting short sleep duration at T3, with the highest risk for chronic short sleep duration (adjusted RR = 2.05).

## Discussion

In short, we found an overall pattern indicating stable or increase in sleep problems from pregnancy to immediately after birth (8 weeks). Whereas the mothers reported relatively fewer sleep problems 2 years postpartum compared to the first two time periods, there was a high stability of insomnia over the three assessment points. Sociodemographical and clinical factors, including concurrent maternal depression could not explain the stability of sleep problems during and immediately after pregnancy, to sleep problems 2 years postpartum.

Sleep duration decreased from pregnancy to the postnatal period from 7 h and 16 min to 6 h and 31 min in the current study. This is in line with a pattern of reduced sleep duration reported by the Hedman study [8]. A similar conclusion was reached by Lee et al. [7] who also found that sleep disturbances were greatest during the first postpartum months. The reduced sleep duration is not surprising given the care demanded by the baby at this time point, including nighttime feeding and the often erratic sleep patterns in newborn babies. Extending these findings, the current study found that the sleep duration had significantly increased to 6 h and 52 min when the child was 2 years old, which is closer to, but still less than the previously reported average sleep duration among Norwegian women aged 40–44 (7 h and 11 mins). A similar pattern was observed for both SOL and WASO. Insomnia showed a high level of chronicity in the present study. While insomnia symptoms increased during the early postpartum period, and declined by 2 years postpartum, the prevalence of insomnia remained very high at year 2 postpartum. Forty-one percent of the women still fulfilled the DSM-IV criteria for an insomnia diagnosis according to the BIS, which is substantially higher than prevalence estimates among women in the same age group (12–17 %) in the general population in Norway [3]. However, when comparing our findings with the validation study of the BIS [21], which included data from women in reproductive age, the differences in insomnia symptoms were smaller. The women in our study reported more nights with non-restorative sleep, and more days with sleep dissatisfaction, while there were only small or no differences on the other BIS items.

Previous studies in the general population have found large age-gender differences when estimating the prevalence of insomnia. For example, whereas a large Norwegian population-based study found few gender differences in young adults, the insomnia prevalence seems to increase markedly from 35 years of age, especially in women, and only to a lesser extent in men [4]. Although few studies have investigated the mechanisms driving such gender differences, it is possible that sleep problems developed during pregnancy may become chronic, and as such contribute to explaining why middle-aged and older women suffer from more sleep disturbances. Future longitudinal studies that track sleep beyond the early years postpartum are needed to explore this hypothesis further. There are, however, other factors which are also likely to explain this gender effect, such has hormonal changes following menopause [29] and gender differences in shift-work occupations [30].

A previous study based on the same dataset as the current study found a close association between insomnia and depression [18], emphasizing the importance of taking depressive symptoms into account when assessing sleep problems in this group of women. However, adjusting for maternal depression did not attenuate the predictive effect of previous maternal sleep problems on neither insomnia status nor short sleep duration at 2 years postpartum. This suggests that the development of chronic sleep problems in this cohort may be largely independent of comorbid postnatal depressive symptoms, despite these conditions being closely interrelated.

The results confirm a rapidly changing pattern of sleep during pregnancy and the first years. Knowledge about the normative sleep pattern among mothers may be important to communicate both realistic expectations in this period, as well as discussing preventive efforts to improve sleep among pregnant women and during early motherhood. Short sleep duration and insomnia are related to a range of impaired functional outcomes, and thus the alterations in sleep may impact the daily life for these women and their children. While some sleep alterations are normative and may be expected, it may be important to prevent chronic sleep problems among new mothers through preventive programs. For some women, the insomnia and sleep problems may be at a level that needs intervention and clinical attention. While treatment for insomnia is efficacious in the general population, tailored interventions may be needed to specifically target pregnant and postpartum women.

The results from the current study must be interpreted in light of several methodological limitations. First, all sleep data were based on self-reported instruments, and not a clinical evaluation or objective measures. Also, while some important confounders were controlled for, other variables that could have influenced the association, such as other maternal psychopathology or medical conditions, were left unexplored. Moreover, the first assessment point in the current study was during pregnancy, and as such, we do not know how many of the woman had suffered from sleep problems or insomnia before they were pregnant. Also, the response rate across all 3 time points was not high, which may limit the generalizability of the sample. Unfortunately, the problem with non-participation in survey research seems to be on the rise [31]. It should also be noted that there were notable differences between the responders and non-responders, with responders being older, more educated, and more likely to be married/cohabitating. Of note, however, no differences in sleep were observed in women who completed all there waves compared to women who dropped out after T1 or T2.

There are several strengths in the present study. The current study is one of the largest studies of sleep during pregnancy, and, to the best of our knowledge, the only prospective study assessing maternal sleep from pregnancy into toddlerhood. As such, it expands on the findings by Hedman et al. [8] who followed 325 pregnant women from 3 months before pregnancy to 3 months after delivery. This may limit the generalizability of the findings. Moreover, this study is, to the best of our knowledge, the largest longitudinal study of sleep problems during and after pregnancy. Also, the questionnaires used in the current study are well-validated instruments, and the BIS has been shown to correspond well with objective sleep measures, including polysomnography (PSG) [21]. And although self-reported sleep parameters, including SOL and WASO typically differ from those obtained from objective assessments [32], recent studies have shown that such self-report sleep assessments can be recommended for the characterization of sleep parameters in both clinical and population-based research [33]. Still, the BIS has not been validated for sleep problems in pregnancy. Similarly, although the EPDS does not provide a clinical diagnosis of depression, it is well suited to assess symptoms of depression among Norwegian postpartum women [24], and the use of the continuous scale (as used in the current study) is also in line with the recommendations for use in population-based research [34].

## Conclusion

In conclusion, both insomnia and short sleep duration were found to be very common both before and after pregnancy. Although the prevalence of insomnia had decreased when the offspring reached toddlerhood, a large proportion of the women still fulfilled the diagnostic criteria for DSM-IV insomnia 2 years postpartum. Given the potential negative impact of maternal sleep problems on later child development, more studies are needed to examine whether early interventions or preventive effort for this group of women may impede the chronic course of sleep problems many of these women experience.

## Abbreviations

BIS: Bergen Insomnia Scale
DSM: Diagnostic and Statistical Manual of Mental Disorders
PSQI: Pittsburgh Sleep Quality Index
SOL: Sleep onset latency
WASO: Wake after sleep onset
EDPS: Edinburgh Postnatal Depression Scale
RR: Relative risk
OR: Odds-ratio
